# Characterization and phylogenetic analysis of the complete mitochondrial genome of *Glossogobius giuris* (Perciformes: Gobiidae)

**DOI:** 10.1080/23802359.2018.1456372

**Published:** 2018-03-26

**Authors:** Pengfei Wang, Chao Zhao, Sigang Fan, LuLu Yan, Lihua Qiu

**Affiliations:** a Key Laboratory of South China Sea Fishery Resources Exploitation & Utilization, Ministry of Agriculture, South China Sea Fisheries Research Institute, Chinese Academy of Fishery Sciences, Guangzhou, PR China;; b Guangdong Provincial Key Laboratory of Fishery Ecology and Environment, Guangzhou, PR China

**Keywords:** Mitochondrial genome, *Glossogobius giuris*, Gobiidae, phylogenetic

## Abstract

The species tank goby (*Glossogobius giuris*) is assessed as Least Concern on the IUCN Red List of Threatened Species. In the present study, we first determined and described the complete mitochondrial genome of *G. giuris*. The circular genome is 16,595 bp in length. It has the typical vertebrate mitochondrial gene arrangement and consists of 13 protein-coding genes, 22 transfer RNA genes, two ribosomal RNA genes, and one non-coding control region. The overall base composition is 29.1% for A, 24.6% for T, 29.4% for C, and 17.0% for G. Phylogenetic analysis of 21 species in the family Gobiidae showed that *G. giuris* was clustered into the genus *Glossogobius*, subfamily Gobiinae. This information will contribute to future phylogenetic studies of Gobiidae and conservation strategies for *G. giuris*.

The species *Glossogobius giuris* is distributed throughout the Indo-west Pacific, and has been assessed as Least Concern on the IUCN red list (Kottelat 2013; Larson et al. 2016). So far, there are no known species-specific conservation measures in place for *G. giuris* (Larson et al. 2016). The mitogenome has been used as an effective tool for molecular taxonomy, species identification, and population genetic analyses in vertebrates (Galtier et al. 2009). However, the mitochondrial genetic information of *G. giuris* is unavailable. Therefore, the complete mitogenome of *G. giuris* was characterized and phylogenetically analysed in this study. We expect that the genomic data will provide essential information to genetic resources conservation and systematic study of *G. giuris*.

The specimen was collected from the Pearl River, Guangdong Province, China. Muscle was sampled and frozen in liquid nitrogen and then stored at −80 °C in South China Sea Fisheries Research Institute (Guangzhou, China). The mitochondrial DNA (mtDNA) was isolated using a Mitochondrial DNA Isolation Kit (Haling Biotech Shanghai, Co., Ltd., Shanghai, China). The whole mtDNA data were sequenced using the Illumina HiSeq Sequencing System (Illumina Inc., San Diego, CA). The clean data were acquired and assembled by the SOAPdenovo Assembler program and GapCloser (Beijing Genomics Institute, Shenzhen, China), and the assembled mtDNA was checked by PCR. The tRNA genes were identified using tRNAscan-SE 2.0 (Lowe and Chan 2016). A maximum likelihood tree of 21 Gobiidae species were constructed using MEGA6.06 software based on the complete mitochondrial genomes (Tamura et al. 2013).

The circular mitogenome of *G. giuris* (GenBank accession number NC_036674) is 16,596 bp in length. It has the typical vertebrate mitochondrial gene arrangement (Wang et al. 2017) and consists of 13 protein-coding genes (PCGs), 22 tRNA genes, two rRNA genes, and a control region (D-loop). Among these genes, *ND6*, *tRNA-Gln*, *tRNA-Ala*, *tRNA-Asn*, *tRNA-Cys*, *tRNA-Tyr*, *tRNA-Ser*^(^*^UCA^*^)^, *tRNA-Glu*, and *tRNA-Pro* are located on the light strand, while all of the remaining genes are located on the heavy strand. The overall base composition is A-29.1%, T-24.6%, C-29.4%, and G-17.0%. All of the PCGs initiate with ATG as start codon, except for COX1, which begins with GTG. This is quite common in vertebrate mtDNA (Yang et al. 2017). Six PCGs (*ND1*, *ND4L, ND6, COX1*, *ATPase 6*, and *ATPase8*) terminate with the typical TAA or TAG as stop codon, while *ND5* ends with AGG. Five PCGs (*ND2*, *COX2*, *COX3*, *ND3*, and *Cyt b*) end with T––, and *ND4* ends with TA–. Twenty-two tRNA genes, ranged in size from 68 to 75 bp, display a typical clover-leaf secondary structure, except for *tRNA-Ser*^(^*^AGC^*^)^. The control region, with high A + T content (62.4%), is located between the *tRNA-Pro* and *tRNA-Phe* genes.

The phylogenetic tree constructed with 21 Gobiidae species (representing five subfamily) clearly demonstrated that *G. giuris*, as well as other Gobiinae species, was supported as a monophyletic group, and *G. giuris* was clustered in the clade of *Glossogobius* species (Figure 1). In all, this genome will contribute to future phylogenetic studies of Gobiidae and conservation strategies for *G. giuris*.

**Figure 1.**
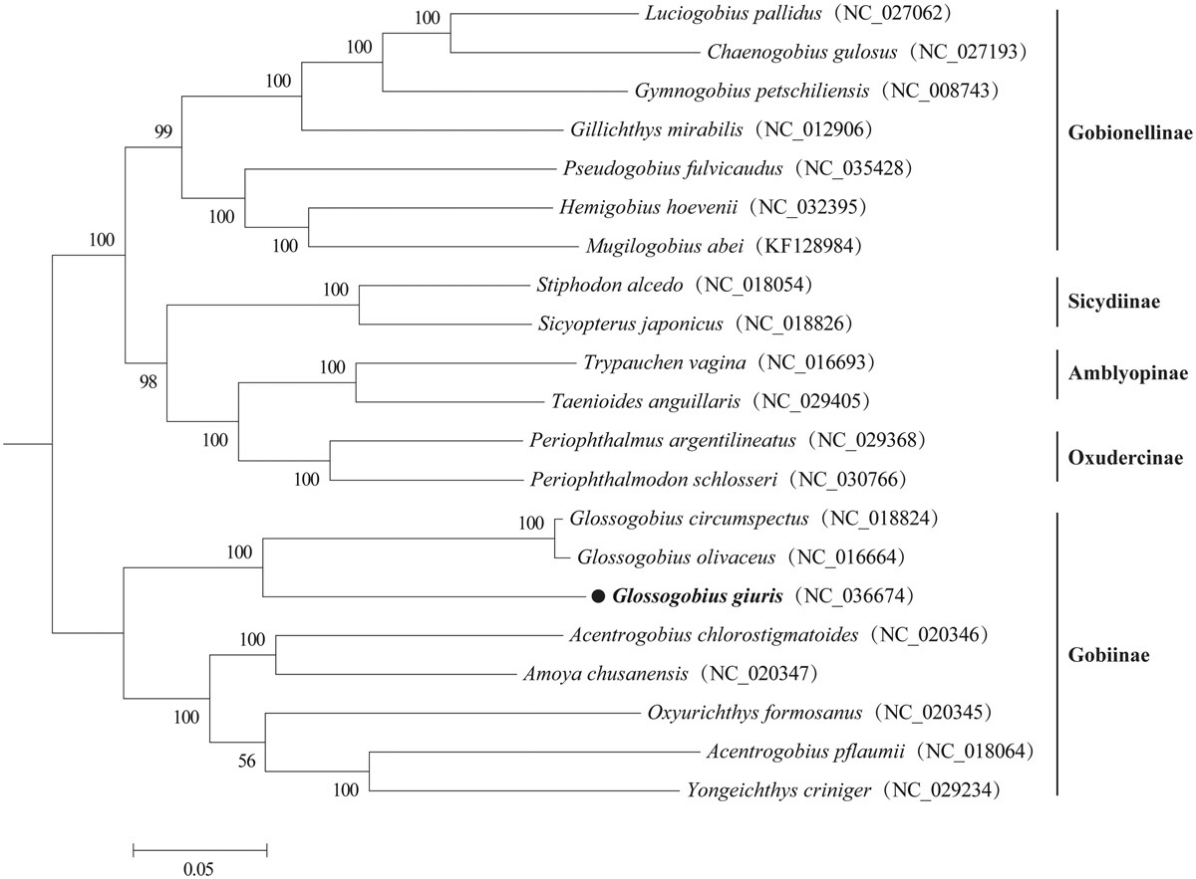
Phylogenetic analysis of 21 Gobiidae species based on their complete mitochondrial genomes using maximum likelihood (ML) method. The tree with the highest log likelihood (180,392.07) is shown. Bootstrap support values (1000 replicates) are indicated at the nodes.
